# Reply: Ongoing under-reporting of clinically relevant safety data in phase II studies of tyrosine kinase inhibitors

**DOI:** 10.1038/sj.bjc.6605629

**Published:** 2010-03-30

**Authors:** S Novello, G V Scagliotti, R Rosell, M A Socinski, J Brahmer, J Atkins, C Pallares, R Burgess, L Tye, P Selaru, E Wang, R Chao, R Govindan

**Affiliations:** 1 University of Turin, Thoracic Oncology Unit, San Luigi Hospital, Regione Gonzole, 10, Orbassano, Turin 10043, Italy; 2 Catalan Institute of Oncology, Barcelona, Spain; 3 University of North Carolina, Chapel Hill, NC, USA; 4 Sidney Kimmel Comprehensive Cancer Center, Johns Hopkins, Cockeysville, MD, USA; 5 Southeastern Medical Oncology Center, Goldsboro, NC, USA; 6 Hospital de San Pablo, Oncology, Barcelona, Spain; 7 Eastern Carolina Internal Medicine, Pollocksville, NC, USA; 8 Pfizer Global Research and Development, La Jolla, CA, USA; 9 Washington University School of Medicine, St Louis, MO, USA

**Sir,**

We acknowledge the discussion points raised by Schöffski *et al* (2010), both in their letter and in their prospective evaluation of sunitinib-induced hypothyroidism published in this journal in 2008 (Wolter *et al*, 2008).

We reported on a small, open-label, phase II clinical trial of sunitinib on a continuous daily dosing schedule in patients with advanced NSCLC and, as noted in the discussion section of the paper, the primary end point of this trial (the reporting of at least five objective responses) was not reached. However, despite the low overall response rate, we were encouraged by the time-to-event results obtained, which we feel are concordant with a signal of activity and with time-to-event data observed on Schedule 4/2 (4 weeks on treatment followed by 2 weeks off; Socinski *et al*, 2008). Furthermore, as this was not a phase III trial designed to definitively assess the activity of sunitinib in patients with lung cancer, we feel justified in reporting intriguing observations pertaining to antitumour activity. Conclusive efficacy statements cannot, and have not, been made in this paper and we believe that the interpretation of the trial data was suitably guarded.

Schöffski *et al* also suggest correlations between the effect of sunitinib on thyroid function and the incidence of fatigue in our trial. There were few data to support such a correlation at the time our trial was designed; consequently, thyroid function measurement was not part of the clinical protocol. Nevertheless, we acknowledge that 53–85% of oncology patients treated with sunitinib develop thyroid test abnormalities, and a substantial percentage develop clinical manifestations of thyroid dysfunction (Desai *et al*, 2006; Rini *et al*, 2007). Guidance for the use of sunitinib in its licensed indications is available and recommends baseline laboratory measurement of thyroid function, with hyper- and hypothyroidism treated as per standard medical practice. Studies investigating the impact of sunitinib therapy on thyroid function are ongoing.
